# Towards the rational design of synthetic cells with prescribed population dynamics

**DOI:** 10.1098/rsif.2012.0280

**Published:** 2012-06-08

**Authors:** Neil Dalchau, Matthew J. Smith, Samuel Martin, James R. Brown, Stephen Emmott, Andrew Phillips

**Affiliations:** 1 Microsoft Research Cambridge, Roger Needham Building, 7 J J Thomson Avenue, Cambridge CB3 0FB, UK; 2 Laboratoire Jean Kuntzmann, Université de Grenoble, B.P. 53, 38041 Grenoble Cedex 9, France; 3 Department of Plant Sciences, University of Cambridge, Downing Street, Cambridge CB2 3EA, UK

## Abstract

The rational design of synthetic cell populations with prescribed behaviours is a long-standing goal of synthetic biology, with the potential to greatly accelerate the development of biotechnological applications in areas ranging from medical research to energy production. Achieving this goal requires well-characterized components, modular implementation strategies, simulation across temporal and spatial scales and automatic compilation of high-level designs to low-level genetic parts that function reliably inside cells. Many of these steps are incomplete or only partially understood, and methods for integrating them within a common design framework have yet to be developed. Here, we address these challenges by developing a prototype framework for designing synthetic cells with prescribed population dynamics. We extend the genetic engineering of cells (GEC) language, originally developed for programming intracellular dynamics, with cell population factors such as cell growth, division and dormancy, together with spatio-temporal simulation methods. As a case study, we use our framework to design synthetic cells with predator–prey interactions that, when simulated, produce complex spatio-temporal behaviours such as travelling waves and spatio-temporal chaos. An analysis of our design reveals that environmental factors such as density-dependent dormancy and reduced extracellular space destabilize the population dynamics and increase the range of genetic variants for which complex spatio-temporal behaviours are possible. Our findings highlight the importance of considering such factors during the design process. We then use our analysis of population dynamics to inform the selection of genetic parts, which could be used to obtain the desired spatio-temporal behaviours. By identifying, integrating and automating key stages of the design process, we provide a computational framework for designing synthetic systems, which could be tested in future laboratory studies.

## 1. Introduction

The field of synthetic biology has the potential to greatly accelerate the development of biotechnological applications, in areas ranging from vaccine development, microbiome engineering and cell therapy [1], to photosynthetic and metabolic production of bio-fuels [2,3]. It could also enable a deeper understanding of fundamental biological design principles [4]. Realizing this potential will require the ability to rationally design populations of synthetic cells with prescribed behaviours, which in turn will require well-characterized components, modular implementation strategies, simulation across temporal and spatial scales and automatic compilation of high-level designs to low-level genetic parts that function reliably inside cells. Many of these steps are incomplete or only partially understood, and methods for integrating them within a common design framework have yet to be developed.

In spite of these challenges, basic genetic devices such as oscillators [5] and toggle switches [6] have so far been implemented at the level of individual cells, while more complex devices such as synchronized oscillators [7], predator–prey systems [8], edge-detection mechanisms [9] and multi-cellular logic circuits [10] have been implemented at the level of cell populations. However, such devices are typically engineered by trial and error, and corresponding models are typically constructed independently, for example, by writing down a set of ordinary differential equations (ODEs). Furthermore, most devices are constructed from only a handful of genetic parts and exhibit relatively simple population dynamics. It has been argued that the design of more complex devices and behaviours will require substantial progress in analysis and modelling tools, together with increased automation [2].

Preliminary steps to automate the design of synthetic biological devices were previously proposed in Pedersen & Phillips [11] through the development of the genetic engineering of cells (GEC) language, a programming language for designing genetic devices. The language allows desired interactions between genetic components of a system to be specified by a programmer, and then infers sets of devices that satisfy the design constraints. The approach relies on a database of genetic parts, characterized by their logical properties and kinetic parameters. The language is *high level* in the sense that the choice of biological parts is left unspecified by the programmer and is instead inferred automatically by a compiler [12]. The approach was illustrated by automatically inferring sets of parts for one of the most complicated genetic designs implemented to date [8], a synthetic predator–prey system. Other examples of high-level languages include GenoCAD [13], which uses formal syntax grammars to constrain the sequences of parts that can be manually selected by a programmer, and Eugene [14], which allows collections of abstract components to be transformed into collections of physical implementations in a design library. The Proto language [15] operates at an even higher level of abstraction, allowing functional behaviours such as logic gates to be compiled to gene regulatory networks. Computational tools and programming abstractions for synthetic biology are reviewed in Purnick & Weiss [16], while languages for biological modelling more generally include Antimony [17], ProMoT [18], iBioSim [19] and little b [20], to name a few. More detailed comparisons between GEC and other languages for synthetic biology are presented in Pedersen & Phillips [11] and Beal *et al.* [12].

In this paper, we present a prototype framework for designing synthetic cells with prescribed population dynamics. The framework extends the GEC language, originally developed for programming intracellular dynamics, with cell population factors such as cell growth, division and dormancy, together with spatio-temporal simulation methods. By using a case study, we investigate how the synthetic predator–prey system of Balagaddé *et al.* [8] could be extended to produce complex spatio-temporal behaviours in future cell colony experiments.

The original design of Balagaddé *et al.* [8] used well-mixed populations of synthetic predator and prey *Escherichia coli* cells grown in a microchemostat, and therefore did not exhibit spatio-temporal behaviour. An extension of this system to cell colony experiments was subsequently implemented in Song *et al.* [21], who investigated the influence of cell motility on simple invasion dynamics. Here, we investigate how more complex spatio-temporal dynamics, such as travelling waves and spatio-temporal chaos, could be produced by a synthetic predator–prey system in Petri dish experiments. We analyse the effects of cell density within the extracellular space, because cells in a Petri dish can grow tightly packed, whereas growth and periodic dilution in a microchemostat can lead to lower densities. Furthermore, we investigate the effect of cells entering a dormant state where nutrients are growth-limiting [22,23]. We consider experiments occurring over much longer time scales than those studied in Song *et al.* [21], to allow for richer spatio-temporal behaviour such as travelling waves [24], spatio-temporal chaos [25] or even stable reaction–diffusion patterns [26]. In so doing, we investigate the extent to which genetic designs at the level of individual cells could influence the behaviour of cell colonies of the order of billions of individuals over multiple generations. Although the results presented here are carried out *in silico*, by basing our design on an existing experimental system, we provide predictions that could be readily tested in future laboratory experiments.

Synthetic systems that produce complex spatio-temporal dynamics have yet to be designed and tested in the laboratory. Our results indicate that designing such systems is non-trivial, and that consideration of multiple factors at different scales is required. Our framework could significantly enhance our ability to design such systems. Complex spatio-temporal dynamics are much-studied phenomena in population biology, which can result from a combination of oscillatory population dynamics and movement of individuals. We have presented an approach that offers, for the first time, the potential to investigate the predictions of theoretical models of spatio-temporal dynamics in synthetic oscillatory systems, through computational simulation and analysis. Our work also highlights the potential for using synthetic experimental microcosms to address fundamental questions in microbial and population ecology, which have traditionally been performed using non-synthetic microbial communities. Example study areas are the maintenance of species diversity in microbial communities [27], microbial population dynamics [28] and spatio-temporal pattern formation [29,30].

The paper is structured as follows. In §2, we present our computational framework for designing synthetic cells with prescribed population dynamics and illustrate our approach by designing a synthetic predator–prey system that, when simulated, produces complex spatio-temporal behaviours. We present an overview of our computational framework (§2.1) together with a summary of our case study (§2.2). We provide a high-level design of the system (§2.3), followed by its automatic compilation to a set of genetic devices (§2.4) and corresponding computational model (§2.5). We then describe how environmental factors are incorporated into the model (§2.6), and explore the range of population dynamics that can be obtained by varying the model parameters (§2.7). Using this information, we constrain the system design to obtain the desired population dynamics (§2.8). Finally, we demonstrate the range of complex spatio-temporal behaviours that are produced by the synthetic design (§2.9). Additional details of the computational methods used in this study are presented in §4.

## 2. Results

### 2.1. Computational design framework

**Figure 1.**
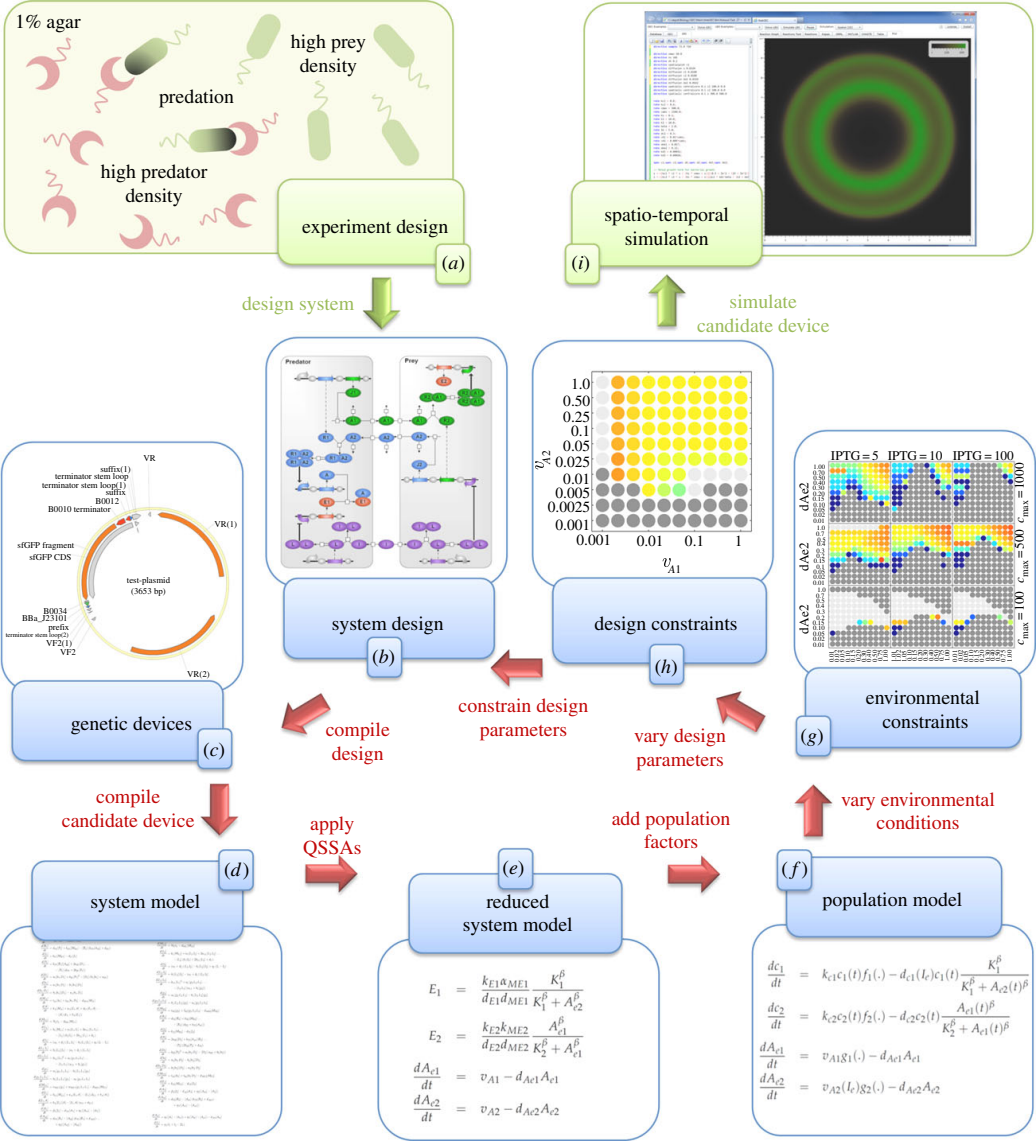
A computational framework for the rational design of synthetic cells with prescribed population dynamics. From a conceptual experiment design (*a*) we program a system design of the genetic device (*b*) using the genetic engineering of cells (GEC) language. Following compilation of the design to a set of genetic devices (*c*; shown as a representative plasmid), we select a candidate device and compile it to a system model (*d*), which characterizes the intracellular dynamics of the device. We then obtain a reduced system model (*e*) as a set of ordinary differential equations (ODEs), under quasi-steady-state assumptions (QSSAs). We then incorporate population factors to obtain a population model (*f*). We conduct parameter scans to identify the environmental conditions that generate the desired population dynamics (*g*). We then vary the parameters of the system to further improve the population dynamics under the chosen environmental conditions (*h*). The parameter scans are then used to constrain the system design, by restricting the kinetic properties of the biological parts that can be selected. We then simulate a candidate device that satisfies the design constraints, using full spatio-temporal simulations (*i*). The candidate device can then be synthesized and tested in the laboratory.

We developed a computational framework for designing synthetic cells with prescribed population dynamics (figure 1), by extending the GEC programming language with cell population factors, such as cell growth, division and dormancy, together with spatio-temporal simulation methods. We start with an *experiment design* (figure 1*a*) involving populations of synthetic cells that interact with each other in a spatial context such as a Petri dish. We then use the GEC language to program a *system design* (figure 1*b*), which specifies the genetic device in terms of desired interactions between genetic parts. The design is then used to automatically generate a set of genetic devices that satisfy the design constraints (figure 1*c*). A candidate device is chosen and compiled to a corresponding *system model* (figure 1*d*), which represents the intracellular dynamics of the chosen device. The model is represented as a set of chemical reactions, which is automatically translated to a set of ODEs, assuming mass action kinetics. A *reduced system model* (figure 1*e*), consisting of a reduced set of ODEs, is then automatically generated, assuming quasi-steady-state dynamics. Further simplifications to the model are made when needed. A set of population factors such as cell growth and division is then introduced, resulting in a *cell population model* (figure 1*f*). Numerical simulations of the cell population model are then produced under different environmental conditions (figure 1*g*), such as different assumptions about cell density and resource limitation. The simulation results are compared with the desired population dynamics, and the most suitable environmental conditions are selected. Refinements to the parameters of the system design are then considered (figure 1*h*), such as modulating the strength of ribosome binding sites (rbs) via base-pair substitutions. The parameter variations are used to expand the range of environmental conditions under which the desired population dynamics are observed, thereby increasing the potential robustness of the system. The system design is then updated to include the additional parameter constraints, to ensure that only genetic devices capable of generating the desired population dynamics are considered. A candidate genetic device is then selected, based on the additional parameter constraints, for simulation in a spatially extended context (figure 1*i*). This enables the resulting spatio-temporal behaviours to be examined under varying initial and boundary conditions. The candidate genetic device could then be synthesized and tested in the laboratory.

In the remainder of this section, we detail each stage of the design process, by considering a case study involving the design of a synthetic predator–prey system that exhibits complex spatio-temporal behaviours.

### 2.2. Programming complex spatio-temporal behaviours in a predator–prey system

Using our design framework, we investigated how the synthetic predator–prey system of Balagaddé *et al.* [8] could be extended to produce complex spatio-temporal behaviours. The original design consisted of two genetic devices, *predator* and *prey*, inserted into two *E. coli* populations grown in a well-mixed setting in a microchemostat. This allowed the population cycles arising from the predator–prey interactions to be modelled using non-spatial population methods. We extended this design by building upon mathematical theory of spatially extended oscillatory systems, which shows that if population cycles are guaranteed in a *well-mixed* setting then spatio-temporal patterns such as travelling waves or spatio-temporal chaos can be observed in a context in which neighbourhood effects are spatially limited [29,31].

Because the model of Balagaddé *et al.* [8] was developed and experimentally tested only for well-mixed systems, we extended this model to take into account spatio-temporal dynamics in a Petri dish environment. The lack of continuous dilution in Petri dish experiments forced us to investigate the effects of a range of density-dependent factors, such as the effects of limited extracellular space on signal concentrations and the effects of limited resources on birth and dormancy or death rates. We explored plausible genetic modifications that could make the system more likely to generate the desired dynamics, and we simulated the final design under different experimental conditions to test these modifications.

### 2.3. Programming the system design

**Figure 2.**
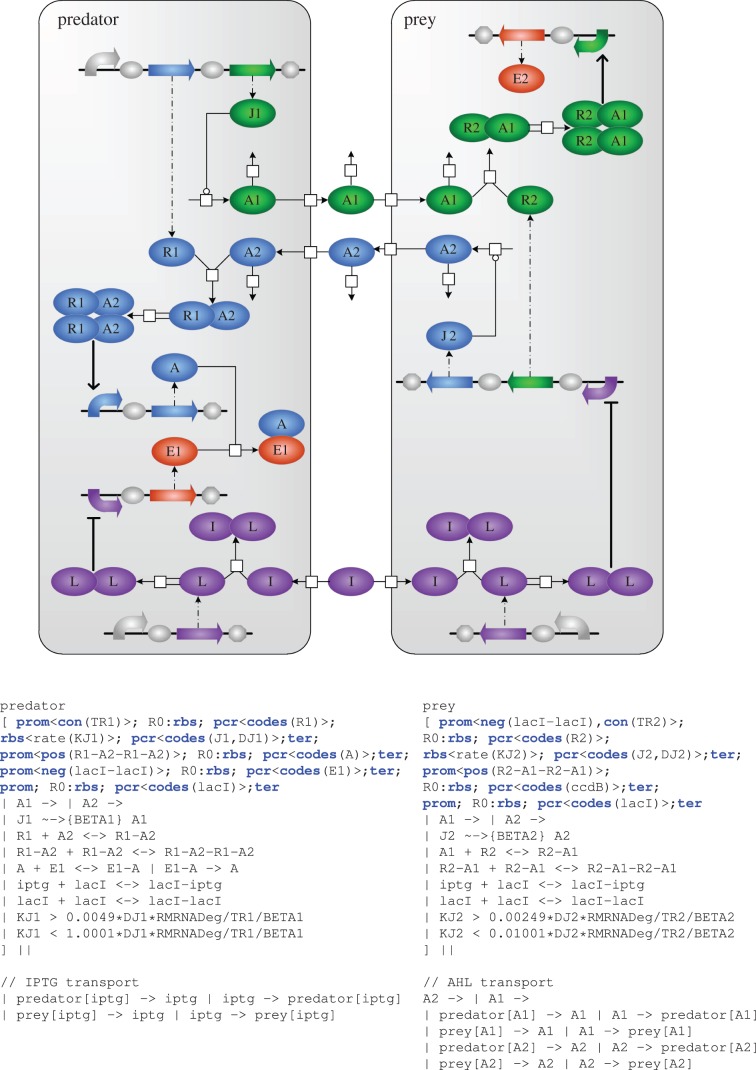
GEC design of the synthetic predator–prey system. Ovals represent proteins and small molecules, square white boxes indicate interactions and genetic parts are represented pictorially according to the definitions of the GEC syntax [11]. The variable names are representative of the components which they seek to describe, as defined in Balagaddé *et al*. [8]: A1 and A2 are AHL molecules, J1/J2 are their synthesizing enzymes and R1/R2 are their receiver proteins, A corresponds to CcdA, E1/E2 is CcdB, I is IPTG and L represents a LacI homodimer.

We programmed the desired behaviour of our synthetic predator–prey system in GEC, a programming language for designing genetic devices [11], in terms of interactions between the molecular components of the system. The main innovation behind GEC is that biological devices can be designed by a programmer with little or no knowledge of the specific genetic parts available. The programmer needs only a basic knowledge of the available part types, namely promoters, rbs, protein-coding regions and terminators. These elementary part types are composed to form system designs, and the desired part properties are expressed as constraints in the GEC language. The full GEC design for the synthetic predator–prey system is presented in figure 2.

In this scheme, the population densities of predator and prey cells depend on the abundance of the MiniF plasmid CcdB (death) protein, which initiates programmed cell death. CcdB is produced in prey cells on receipt of an intercellular signal generated by the predator cells. It is produced constitutively inside the predator cell, and an antidote protein (CcdA) is induced in the predators upon receipt of a signal from the prey. In this way, the prey are needed to ensure the survival of the predators.

Each cell type recognizes the presence of the other using synthetic intercellular signalling devices, derived from natural quorum-sensing components. The predator cells produce a distinct acyl homoserine lactone (AHL) 3-oxo-C_12_-HSL (AHL12), which binds to the LasR transcriptional activator within the prey cells and initiates transcription of CcdB. The prey cells produce a second AHL, 3-oxo-C_6_-HSL (AHL6), which binds to the LuxR transcriptional activator and initiates transcription of the CcdA antidote protein in the predator cells. The CcdB lysis protein that causes cell death was placed under the control of a LacI-inducible promoter in the predator cells to modulate downstream gene expression. The activity of the constitutively expressed LacI-regulated promoter is modulated by supplying isopropyl **β**-d-1-thiogalactopyranoside (IPTG), which binds to LacI dimers, preventing formation of transcription-initiating tetramers.

The GEC code was more detailed than in [11] because the new model required (i) explicit dimerization of AHL-receiver for downstream transcriptional induction and (ii) that the P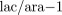 promoter is explicitly induced by LacI molecules, with IPTG blocking the tetramerization of two LacI homodimers [32] (figure 2).

### 2.4. Automatically deriving the genetic parts

The GEC language allows a user to program the desired behaviour of a genetic device, and to infer candidate implementations by searching a database of well-characterized biological parts [11]. Compiling the GEC program initiates a search over the database, the result of which is a set of all solutions that satisfy the design constraints. Upon selection of a particular solution, the GEC software produces a model of the intracellular behaviour of the device, in the form of a set of biochemical reactions, with rate constants as defined in the database. The reactions are displayed in the language for biological systems (LBS; [33]). Compilation of LBS code permits stochastic simulations to be carried out using the Gillespie algorithm, and deterministic simulations using a Runge–Kutta–Fehlberg ODE solving routine. The compilation also produces a series of representations of the LBS code, including a graphical representation and a Matlab function that can simulate the reactions as a system of ODEs.

To enable GEC to find solutions that matched the desired topology, we created minimal databases of biological parts and reactions (detailed in the electronic supplementary material, tables S2 and S3, respectively). As the program specified a total of nine rbs and our database contained two different rbs parts, compilation of the program resulted in a large number of solutions. Taking into account the two different ways of assigning AHL molecules to predator and prey cells, GEC returned 2 × 2^9^ = 1024 solutions. The multiplicity of solutions illustrates both the flexibility and uncertainty conferred to the designer when constructing a synthetic biological device. These different GEC solutions are described by the same set of system reactions, with only the kinetic rate parameters being variable. Therefore, to analyse the system design, we selected the first solution from the list and generated the corresponding reaction network, together with a set of ODEs in Matlab code (see the electronic supplementary material, A.1 and A.2, for the full reaction network and ODE model).

### 2.5. Automatically deriving the system model

Model reduction techniques were applied to the system model derived by GEC (figure 1*d*), producing equations that are more amenable to quantitative analysis (figure 1*e*). To do this, we systematically applied quasi-steady-state assumptions (QSSAs), by assuming that the kinetics of some reactants operated on a faster time scale than the kinetics of others, such that the reactants were effectively in dynamical equilibrium. We made this assumption for all intracellular reactions, allowing the system to be formulated purely in terms of the extracellular concentrations of the AHLs (see §4 and the electronic supplementary material, A3, for details). This form of model reduction was also used by Balagaddé *et al.* [8] to analyse the optimal concentration of IPTG for generating population cycles.

When we compared the equations in Balagaddé *et al.* [8] with those derived for our model, we found two differences. First, our model indicated that the parameter *K*_2_ (half saturation constant for the functional response of prey cells to AHL6) was IPTG-dependent (in addition to 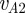 and *d*_*c*1_). As the receiver protein LasR is produced downstream of the P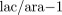 promoter, which is IPTG-activatable, we conclude that the analysis in Balagaddé *et al.* [8] claiming that *K*_2_ is not IPTG-dependent cannot be justified. The second difference arose in the functional form of the IPTG-dependent parameters. In Balagaddé *et al.* [8], a second-order Hill function was assumed, whereas we derived a slightly more complicated functional form from a plausible reaction set involving LacI, IPTG and 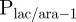. Realistically, the difference in these functional forms is likely to confer only minor effects on the dynamics of the system, as the leading order of both the numerator and denominator is quadratic in the concentration of IPTG. Nevertheless, the fact that differences can be identified illustrates an advantage of automating the design procedure. It is also possible that automation could obfuscate poorly justified assumptions, such as in the application of the QSSAs [34]; so it would be important to incorporate rigorous checks for their validity.

The reduction of the model equations can be achieved in a semi-automated way. After selecting which concentrations are to be dynamic, the derivatives of the remaining concentrations can be equated to 0 using software with an algebraic solving capability, such as Mathematica or Maple. Equations representing gene regulatory networks often produce characteristic functional forms, such as the Hill function. Furthermore, each concentration usually depends on no more than three upstream variables, simplifying the form of the final reduced equations (figure 1*e*). Consequently, as was observed in this case, applying QSSAs can lead to a very simple set of equations. The combination of the equations for the steady-state and dynamic concentrations provides a complete approximate representation of the synthetic circuits in each cell type.

### 2.6. Programming the cell population model

By producing a reduced equation set that incorporates the environmental and population-level factors, it is possible to rapidly assess the impacts of the environment on the performance of the device (figure 1*g*). In this section, we illustrate how we incorporated and analysed alternative assumptions about environmental factors in the simplified cell equations.

The key difference between the microchemostat and Petri dish experiments is that populations could potentially rise to higher densities in Petri dishes. Unlike microchemostats, continuous dilution is not possible, and *E. coli* populations can grow until resources are limiting or until other density-dependent factors (e.g. toxins) build up to such an extent that the populations stop growing. To incorporate these effects, we re-derived the population model of Balagaddé *et al.* [8] from their set of biochemical reactions, considering whether any of their calculations required making assumptions about physical space or resources not becoming limiting (see the electronic supplementary material, B, for details).

The first modification we made to the model of Balagaddé *et al.* [8] was to remove the effects of dilution. Second, we relaxed the assumption that the ratio of the volume of extracellular space to the space occupied by *E. coli* is high, an assumption likely to be violated in dense populations ([35], details given later). Third, resource availability is likely to become both limiting and spatio-temporally heterogeneous in Petri dishes [21,30]; so we represented resources explicitly. Next, we considered negative density-dependent effects on population growth. Such effects are commonly abstractly incorporated as an increasing or constant *per capita* mortality rate as a function of density [29,30,36]. However, an increasing death rate is not generally observed over the relatively short time scales considered in our study (days to weeks). Rather, cells enter a dormant state, in which metabolic activity is considerably reduced. Cells can potentially recover from their dormant state when resources become available again [22,23], though, in controlled experiments, this effect is most likely to be negligible. We therefore explored the effects of incorporating either density-induced mortality or dormancy to cover both of these eventualities (details given later).

Taking our considerations of the effects of limiting space and resources into account led us to derive the following form for the population model:
2.1a
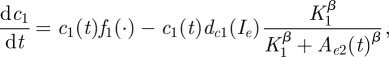

2.1b
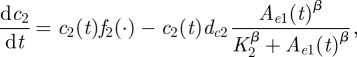

2.1c
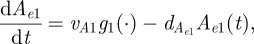

2.1d
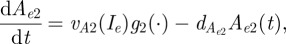

2.1e
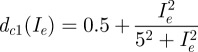

2.1f
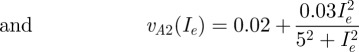

Here, *c*_1_ and *c*_2_ are the population densities of active predators and prey (cells ml^−1^), respectively, and *t* is time (hours). *A*_*e*1_ is the concentration of an ‘antidote’ signal, AHL6 (nM), in the extracellular space with decay rate 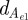 (h^−1^), and *A*_*e*2_ is the concentration of a ‘killer’ signal, AHL12 (nM), with decay rate 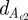 (h^−1^). *K*_1_ and *K*_2_ are half saturation constants (nM), and **β** scales the shape of the death rate functional responses (dimensionless). *d*_*c*1_ and *d*_*c*2_ are the death rates (h^−1^) and 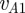 and 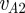 are the production rates for the AHLs (nM h^−1^). As in Balagaddé *et al.* [8], *d*_*c*1_ and 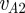 vary with the extracellular concentration of [IPTG] (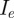, µM) according to (2.1e) and (2.1f), respectively. We use 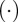 to denote a generic function representing an assumed effect of the environment or population factor. In this case, 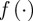 and 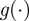 are always non-negative (described separately in the following subsections).

#### 2.6.1. Effect of cell density on signal production

As the population density of *E. coli* increases in a Petri dish towards an absolute maximum density 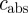, the volume of extracellular space in the medium decreases and can no longer be considered much larger than the space occupied by the *E. coli*. To incorporate this effect on the concentration of AHLs in the extracellular medium, we derived an alternative functional form for 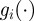,
2.2
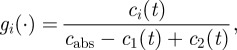

where *i* = (1, 2). Here, 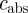 is defined as the absolute maximum packing density for typical *E. coli*, which we estimated to be approximately 1540 cells pl^−1^ (see §4 for details), although this would not be achieved in practice owing to resource limitations and packing constraints. The effect of modifying this function is that the rate of increase of the concentration of a given AHL in the medium, for every quantity of AHL emitted per individual *E. coli* into that medium, becomes higher because the extracellular volume within which it is diluted becomes less. Note that for comparison with the original model, where *g*_*i*_ = *c*_*i*_, we need to rescale 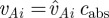, with 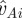 taking the values in Balagaddé *et al.* [8].

#### 2.6.2. Effect of resource limitation on population growth and CcdB-independent mortality

To explicitly incorporate the effects of resource limitation on the *E. coli* birth rates, we altered the *per capita* population growth rate function *f*_*i*_ from the logistic form used by Balagaddé *et al.* [8] to
2.3
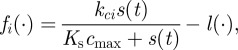

where *i* = (1, 2). For simplicity, we treat resource availability *s* as having the same units as predator and prey (cells pl^−1^), and 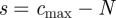, where *N* is the total abundance of *E. coli*. In this context, 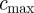 is the maximum carrying capacity as determined by total resource availability. 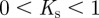 is the half saturation constant of the birth rate function, normalized to be a constant fraction of the carrying capacity, and for simplicity we assume 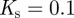 because it results in a functional form that is similar to the logistic equation assumed by Balagaddé *et al.* [8]. We considered two alternative formulations for the CcdB-independent mortality/dormancy rate function 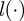. The first of these makes the standard assumption of a density-dependent mortality rate
2.4
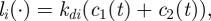

where *i* = (1, 2). To estimate the mortality rate 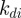, we assume that, in the complete absence of nutrient limitation, the death rate owing to the build-up of toxins will balance the birth rate when 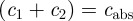 (i.e. at the absolute maximum population density). This leads us to estimate that 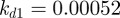 and 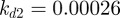. For simplicity, we also assume that nutrients are immediately returned to the medium when cells die [37]. Consequently,

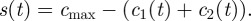



#### 2.6.3. Effect of resource limitation on population dormancy

To explore the effects of dormancy in increasingly dense cell populations, we considered using 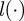 in (2.4) to instead represent the rate of conversion of individuals to a pool of dormant individuals. Losses owing to dormancy are different from mortality in three key respects: (i) the nutrients absorbed by dormant individuals remain unavailable to the active cell populations, (ii) dormant individuals have an effect on the availability of extracellular space, and (iii) the death rate of dormant individuals will still be a function of the relevant concentration of AHL in the medium. We modelled these effects by adding equations to represent the populations of dormant predators and prey
2.5a
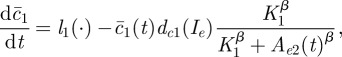

2.5b
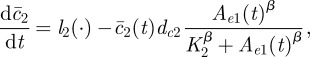

2.5c
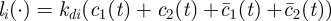

2.5d
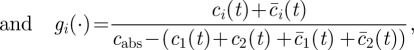

where *i* = (1, 2) and 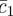 and 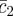 are the population densities of dormant predators and prey, respectively. For simplicity, we assume that dormant cells retain the ability to produce AHL molecules and that their mortality rate is a function of the external AHL concentration. Therefore, the only difference between dormant and non-dormant individuals is that dormant individuals cannot reproduce. Finally, we assume that nutrient availability follows

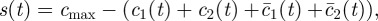

consistent with the definition given earlier.

### 2.7. Constraining the environmental conditions

**Figure 3.**
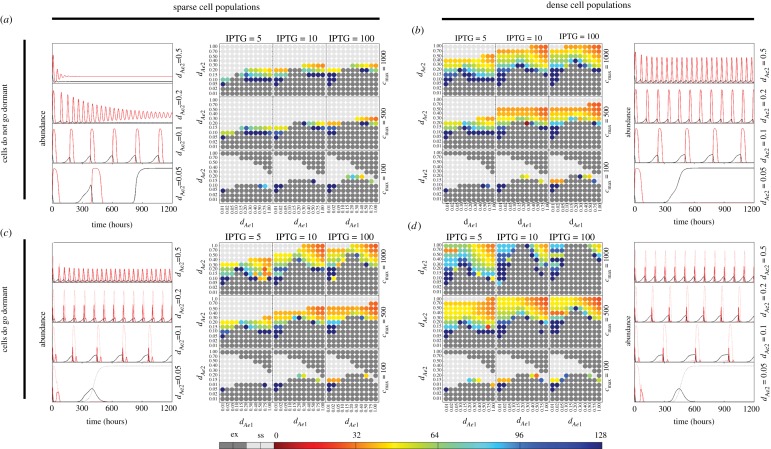
Effects of environmental factors on cyclic dynamics of the synthetic predator–prey system. The synthetic predator–prey system model was simulated under varying environmental conditions, assuming cells never go dormant (*a*,*b*) or can go dormant when nutrients are limiting (*c*,*d*). Also compared are the dynamics corresponding with sparse populations (equivalent to a microchemostat; *a*,*c*) and dense cell populations (equivalent to a Petri dish; *b*,*d*). In each quadrant, the parameters *I*_*e*_, 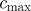, 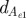 and 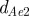 were varied to assess the impact of density dependence on the dynamics of populations of predator and prey cells. The qualitative nature of the dynamics is characterized by whether the populations coexist stably (light grey spots), oscillate (coloured spots corresponding to period, see colour scale) or one population dominates leading to extinction of the other (dark grey spots). Additionally, representative simulations are performed for *I*_*e*_ = 10 µM, 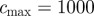 ml^−1^, 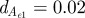 h^−1^ and 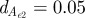, 0.1, 0.2, 0.5 h^−1^, illustrating the population of active predators (solid red lines), active prey (solid black lines), dormant predators (dotted red lines) and dormant prey (dotted black lines).

All of the formulations of the non-spatial population dynamics equations required sufficiently high IPTG concentrations to predict population cycles for realistic parameter values (figures 1*g* and 3). Low IPTG concentrations resulted in slower variations in the expression of the genes controlling the predator death rate and antidote production rates. This resulted in population dynamics that converged to a stable steady state over time. As a result, we restricted our attention to simulations in which IPTG ≥ 5 µM.

We compared the predictions of our population dynamics model under different combinations of assumptions about density dependence, volumetric effects and parameter values (figure 3). In all scenarios, low antidote decay rates resulted in predator extinction. A detailed analysis of the bifurcation structure revealed that these were in fact extremely long period population cycles in which predator densities were driven to very low levels for very long periods of time (see electronic supplementary material, figure S1). We classified these dynamics as ‘predator extinction’ because the lengths of these cycles, and possibly implausibly low population densities, are likely to be impractical for laboratory experiments. As the antidote decay rate 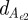 was reduced from relatively high rates, we observed a transition in the predicted dynamics from an equilibrium steady state, to periodic oscillations, to predator extinction (see figure 3 and electronic supplementary material, S1).

When 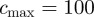 ml^−1^ and the antidote decay rate 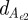 is sufficiently high, then the predator populations can be driven to extinction. Intuitively, this can happen if the decay rates of antidote are so high that there is never sufficient antidote to maintain a positive population growth rate. In cases in which the carrying capacity is sufficiently low, the predator population can never rise to a sufficient abundance to produce sufficient antidote to permit a positive growth rate in the predator population, and the predator population goes extinct. Mathematical and numerical analysis confirms the absence of a stable coexistence steady state for sufficiently high antidote decay rates (see the electronic supplementary material for details).

Increasing the carrying capacity from 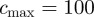 ml^−1^ resulted in a wider range of antidote and killer decay rates that led to population cycles (figure 3). It is widely known that increasing the carrying capacity in coupled models of trophic interactions can lead to a destabilization of the homogeneous steady state to periodic oscillations: this is the classic ‘paradox of enrichment’ [38]. Our observations are another example of that phenomenon; increasing the carrying capacity can lead to a transition in the predicted dynamics from a stable coexistence steady state to periodic oscillations.

Assuming density-dependent mortality generally leads to population cycles being predicted for a relatively narrow range of parameter values (figure 3*a*). The range of 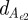 is expanded only slightly by increasing 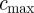. This is generally the pattern predicted for the equations of Balagaddé *et al.* [8] for the same range of parameter values but without dilution. If instead we assume that individuals do not die, but enter a dormant state, then the range of parameter values predicting population cycles increases (figure 3*b* and electronic supplementary material, S1). Dormancy limits the population growth rate by not removing non-reproductive individuals, leading to increased negative density dependence. The reduced *per capita* birth rates result in deeper population crashes, which increase the time lag between predator and prey population responses and destabilize the population dynamics for a wider range of parameter values than in the absence of dormancy effects.

The assumption of the extracellular volume being limited (without density-dependent dormancy) also leads to an expansion of the parameter region predicting population cycles, especially at high 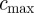 (figure 3*c*). Adding the volume limitation effects on AHL production rates results in an increased *per capita* production rate of killer and antidote molecules, which in turn results in more marked population crashes. The resulting increased time lag in population responses is sufficient to destabilize the population dynamics. The time-lag effects of dormancy and volume-dependent AHL production appear to be complementary, resulting in further expansion of the region of parameter space predicting population cycles.

### 2.8. Constraining the system design

The previous analysis into the effects of the environment, IPTG addition and density-dependent dormancy on the propensity of the synthetic predator–prey system to exhibit population cycles revealed some flexibility in the environmental parameters that could be chosen to result in the desired behaviour. At this stage, one could attempt to obtain numerical estimates of the environmental parameters and deduce from figure 3 whether cycles are likely to occur, and the characteristics of those cycles. In the situation that cycles are not present, or that the oscillation period is too long, it could be desirable to modify the original genetic design. In our quantitative framework, this turns out to be a simple extension of the environmental parameter scan in figure 3 (as shown in figure 1*h*).

By applying single base-pair mutations to specific promoter or rbs sequences, it is possible to generate libraries of diversified components that have slightly differing quantitative characteristics [39,40]. In the case of rbs elements, numerical algorithms offer approximations of such sequence manipulations [40]. As an example of how to refine designs of synthetic biological systems, we considered modulating the rate of AHL synthesis by changing the strength of the rbs elements that lie upstream of the coding sequences for LuxI and LasI, the enzymes that catalyse the production of AHL6 and AHL12 (figure 1*h*). In general, the rate of translation depends on both the rbs and the 5′ end of the coding sequences. However, recent work has shown that spacer sequences can be inserted such that protein expression levels correlate with rbs strength with high precision [41]. Therefore, we proceed by assuming that increasing or decreasing the strength of rbs sequences is analogous to modifying the rates of AHL synthesis, 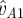 and 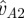.

**Figure 4.**
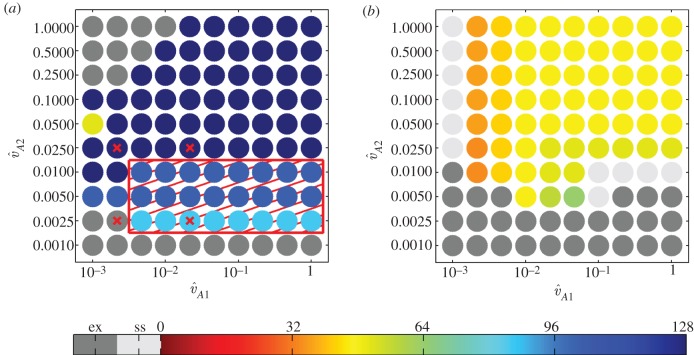
Selection of optimal ribosome-binding site (rbs) strengths. Oscillation period was calculated as a function of the AHL activation constants 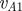 and 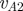 in the model with high-density populations and density-dependent dormancy, to assess how to optimally assign rbs in the genetic design. The two cases presented correspond to the parameter set used in Balagaddé *et al*. [8] with [IPTG] =5 µM and 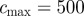 ml^−1^, and the AHL degradation rates set to (*a*) 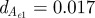 h^−1^, 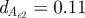 h^−1^ and (*b*) 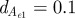 h^−1^, 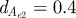 h^−1^. In (*a*), the red hatched box indicates a desirable region, while the red crosses indicate the values of 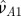 and 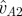 that are possible from the parts database in the electronic supplementary material, table S2. Note that only one pair of values lies within the desirable region.

We varied 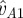 and 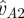 over a wide range of values to select the optimal rbs. Initially, we did this for the parameter set used by Balagaddé *et al.* [8], which led to the prediction that 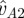 can be assigned optimally near 0.0025 nM h^−1^ provided that 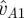 exceeds approximately 0.05 nM h^−1^ (figure 4*a*). Afterwards, we considered the case where the pH of the growth medium was buffered such that the degradation rates of the AHLs were increased to 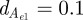 h^−1^, 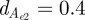 h^−1^ (figure 4*b*). In contrast to the unmodified AHL degradation rates in figure 4*a*, we found an opposite qualitative dependency on the AHL synthesis rates, as 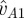 could be assigned an optimal value (near 0.0025 nM h^−1^) provided that 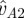 was no less than 0.01 nM h^−1^. As expected, the oscillation period was considerably reduced when increasing the degradation rates with pH buffers (figure 4).

We used the GEC software to impose conditions on kinetic rates associated with specific interactions, allowing us to assess conditions under which the desired behaviours are predicted to occur, and to specifically use biological parts that adhere to these conditions. In relation to our synthetic predator–prey case study, we sought to incorporate the analysis of figure 4 into the GEC code. From the definitions of the simplified model parameters 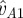, 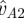, *K*_1_, *K*_2_, *d*_*c*1_ and 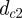 in terms of the rates of the underlying chemical reactions (see the electronic supplementary material, table S1), we found that 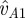 and 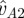 could be manipulated independently of the other simplified model parameters via changes in the rates of translation or degradation of the AHL synthesizing enzymes LuxI and LasI, *k*_*J*1_, *k*_*J*2_, *d*_*J*1_ and *d*_*J*2_. Under the assumption that the rate of translation can be set independent of the coding sequence, we focus on how to select optimal values for *k*_*J*1_ and *k*_*J*2_. The strength of rbs can be constrained directly by GEC. As an example, we use the constraints in figure 4*a*, whereby we require 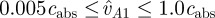, 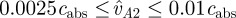. This is equivalent to requiring

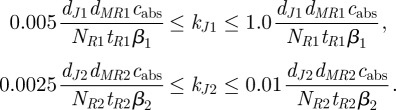

Adding these inequalities into the GEC program is achieved by assigning parameters to specific parts and writing the inequalities in terms of these parameters. For example, the rbs elements associated with *J*1 and *J*2 synthesis can be assigned rates as rbs<rate(KJ1)> and rbs<rate(KJ2)>, while constitutive promoters can be written as prom<con(TR)>. To further constrain the number of solutions, we imposed a constraint that all remaining rbs elements use the same part, by assigning a variable R0 (written R0:rbs). Finally, by default, GEC assumes that the gene copy number is 1; for illustrative purposes, we left this unchanged (i.e. 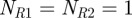). This results in a revised GEC program that, when compiled, results in only four solutions (electronic supplementary material, section D, gives details). Both the rbs elements associated with AHL synthesizing enzymes were equal to the database entry rbs2 in all solutions, while R0 was assigned as either rbs1 or rbs2 and A1/A2 was assigned interchangeably as AHL6/AHL12. The values of 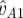 and 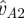 that are achievable from the database (detailed in the electronic supplementary material, tables S2 and S3) can be visualized in figure 4.

### 2.9. Simulating spatio-temporal behaviour

**Figure 5.**
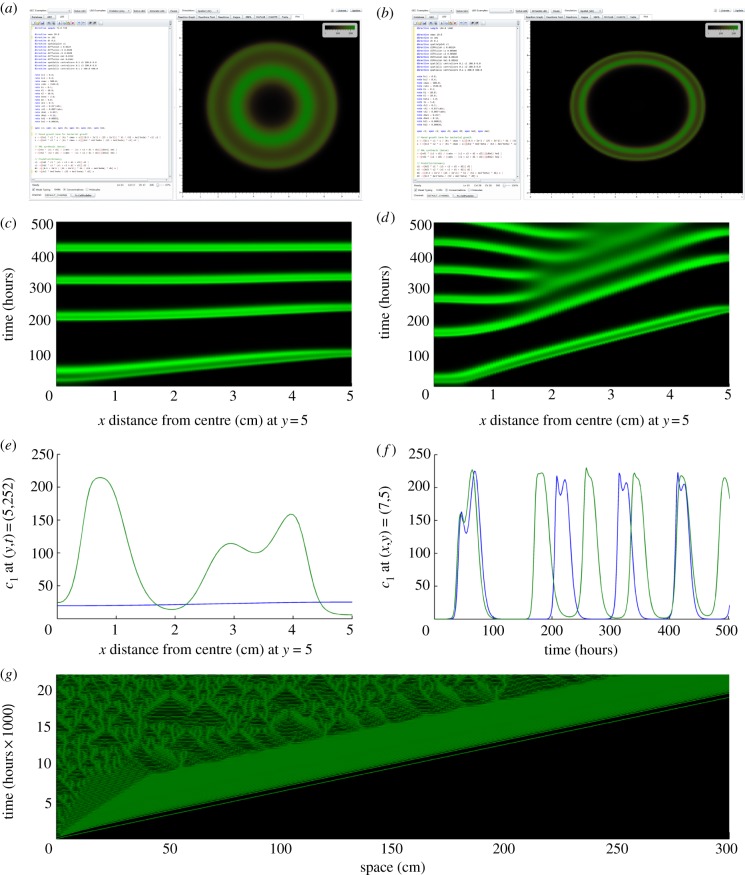
An invasion scenario yields irregular travelling waves in an optimized synthetic predator–prey system. (*a*,*b*) Snapshots of simulated predator density (*c*_1_, cells pl^−1^) within the GEC tool are displayed for two different simulations of the reaction–diffusion equations (2.6) (*c*,*d*) Space–time plots of predicted dynamics at *y* = 5 and *x* between 5 and 10 for 500 simulated hours (about three weeks) for (*a*,*b*), respectively. Both simulations started with *c*_1_ = 100 cells pl^−1^ and *c*_2_ = 100 cells pl^−1^ in the central 0.5 cm× 0.5 cm of the simulated environment (*s* = 300 cells pl^−1^) and *c*_1_ = *c*_2_ = 0 cells pl^−1^ everywhere else 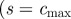). (*a*,*c*) Predators and prey diffuse at 0.0108 cm^2^ h^−1^, nutrients and AHL6 diffuse at the faster rate of 0.0324 cm^2^ h^−1^, and AHL12 diffuses at half the rate of AHL6 because it is a larger molecule (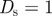, where 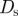 scales all diffusion rates; see the electronic supplementary material, table S1). The snap-shot was taken after 3 simulated days. (*b*,*d*) The diffusion coefficients are exactly an order of magnitude smaller than those in (*a*) (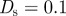). The snap-shot was taken after 6 simulated days. The same set of parameter values was used in both simulations (other than the diffusion coefficients), chosen from the results in figure 4*b*, with 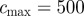 cells pl^−1^, 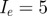 µM, 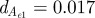 h^−1^, 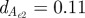 h^−1^, 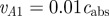 and 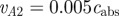 (see the electronic supplementary material, table S1, for other parameter values). We simulated the partial differential equations with GEC (see the electronic supplementary material, section E), which uses a semi-implicit finite difference method based on the Crank–Nicolson method [42], with space discretization of 0.1 cm and a time step of 0.1 hours. The colour scale ranges from black to green, with black corresponding to low predator density and green being high predator density. (*e*) Predator density as a function of space at one moment in time, and (*f*) as a function of time for one point in space, for the two invasion scenarios shown in (*a*–*d*). (*g*) Predicted dynamics using the same equations and parameters as in (*b*) and (*d*) but assuming one-dimensional space, a much larger domain (we used a 1000 cm domain length but we only show 300 cm here), and longer simulated time (up to 22 000 hours is shown here). (*e*,*f*) Blue line, 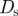 = 1; green line, 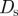 = 0.1.

Having completed a full design cycle, understanding how to combine the effects of a variety of environmental and population-level factors with specific biological parts, we investigated how this translated to spatio-temporal dynamics in simulated Petri dish experiments (figures 1*i* and 5).

In the absence of chemotaxis (assumed here for simplicity, though this could be enforced by using non-chemotactic *E. coli* mutants), such spatio-temporal dynamics can be simulated using reaction–diffusion equations of the form
2.6a
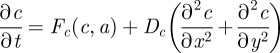

and
2.6b
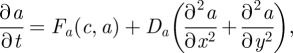

where *c* = *c*(*x,y,t*) is the vector of cell densities, *a* =*a*(*x, y, t*) is the vector of extracellular signalling molecule concentrations, 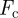 represents the interactions between the cells and molecules that influence the growth or decline of the cell populations and 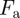 represents the interactions that influence the kinetics of the molecules. The diffusion rates of each species are represented by 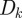 (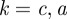), while *x* and *y* represent the spatial dimensions of the Petri dish.

We used the results of the parameter scans (figure 4) to select a model formulation and a set of parameter values for simulating the reaction–diffusion equations (2.6). For the interactions term, we chose what we believe to be a realistic model formulation, incorporating density-dependent dormancy and volumetric effects on AHL production (i.e. equations (2.1) and (2.5) with functional forms (2.2)–(2.4)). Parameter values were sought that generated population cycles with a relatively short temporal period, and we chose parameters corresponding to the redesigned system analysed in figure 4*b* (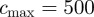 ml^−1^, 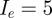 µM, 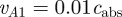 and 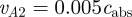). We chose two sets of diffusion rates, one set based on values in Tyson *et al.* [36] (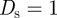, where 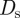 scales all diffusion rates; see the electronic supplementary material, table S1) and another set with the same values reduced by an order of magnitude to simulate the effects of increasing the thickness of the nutrient agar (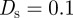). An ‘invasion scenario’ (which is perhaps more realistically termed a ‘colonization’ scenario but we adopt the standard terminology here) was simulated in which equal densities of predator and prey are placed in a central core of the Petri dish with only nutrient in the surrounding region.

Using our selected parameter values, the invasion scenario predicts a moving invasive front from the centre to the edge of the Petri dish over a period of around 5 days in the faster diffusion scenario (figure 5*a*,*c*,*e*,*f*) and 10 days in the slower diffusion scenario (figure 5*b*,*d*–*f*). Detailed inspection of the spatio-temporal dynamics over 504 simulated hours (three weeks) indicates that the invasion front in the faster diffusion scenario traverses the Petri dish before a new wave begins in the centre (figure 5*c*). These dynamics then clearly begin to converge towards spatially homogeneous oscillations (figure 5*c*,*e*). In contrast, a few irregular travelling waves are generated behind the invasion front in the slower diffusion scenario over the same period (figure 5*d*–*f*). Note that these are not ‘periodic travelling waves’ in the strict mathematical sense, which are one-dimensional travelling waveforms of constant speed and shape [24]. Simulating the same equations with the slower diffusion rates on a much larger one-dimensional domain (we only show a selected region in figure 5*g*) reveals the phenomenon of ‘dynamical stabilization’ behind the invasion front [43], in which the reactants approach, and remain close to, their unstable coexistence steady state behind the invasion front for a period of time before transitioning to what appears to be spatio-temporal chaos (though we have not confirmed whether it is indeed spatio-temporal chaos). Intriguingly, the region exhibiting dynamical stabilization in this simulation reaches an approximately constant width (about 50 cm), which phenomenologically appears to be very similar to the constant-width band of periodic travelling waves observed behind invasion fronts in studies of other oscillatory reaction–diffusion systems (e.g. fig. 1 of [25]). Note, however, that the domain size (metres) and simulation length (years) we used is practically infeasible, and further design modifications would be needed to obtain such dynamics for more practical experiments.

## 3. Discussion

Automating the design of synthetic biological devices is a long-term goal in the field of synthetic biology, which could enable significant advances in biotechnological applications. In this article, we have presented a computational methodology to aid the design of synthetic cells with prescribed population dynamics, which proposes biological parts most likely to achieve the desired behaviour (figure 1). The GEC software solves a constraint problem to select genetic parts that behave according to specification, though it requires accurate knowledge of the behaviour and kinetics of each part. Currently, few individual parts have been well characterized, with the best example of characterization being at the level of *devices* consisting of combinations of parts, such as the 3-oxo-C_6_-HSL receiver system [44]. A database of characterized parts is a useful resource, provided that the interactions between the parts of a candidate design are rigorously tested, for instance, to check for potential cross-reactivities between transcription factors and promoters, or to determine the concentration of transcription factors needed to saturate a given promoter. Another issue relates to the multiplicity of solutions. In this study, we investigated multiple solutions by considering a parameterized model that represented the set of all possible solutions. We then used this model to generate constraints on specific genetic parts, using parameter variation methods for combinations of parameters (figure 4). In some cases, the constraints may suggest using parts that are not yet in the database, such as the creation of stronger rbs elements.

Several technical challenges limit the use of software in automating the design of synthetic biological systems. Most of the software tools assume that the interactions between specific promoters and regulators are known, and that the kinetics of all DNA elements have been well characterized. Furthermore, it is often assumed that the synthetic circuit can operate independently from the host cell, despite the clear problem that high expression of the synthetic circuit will undoubtedly impinge on the resources of the cell, reducing its overall fitness and therefore limiting the output of the synthetic device. Despite these limitations, the development of software aimed at characterizing what is possible to create is still of importance.

We have shown how the full model of Balagaddé *et al.* [8] can be derived automatically from an initial component-level design, whereas the original model was constructed entirely by hand. We have also used partial automation to perform model reduction, allowing efficient simulation of long-running spatio-temporal dynamics. Our tools for model derivation and reduction demonstrate how a number of inconsistencies in the original, manually derived model can be detected and fixed.

The reduction of models to fewer equations using QSSAs is standard practice, though typically involves subjective judgement [34], such as determining which concentrations operate on fast versus slow time scales. We implemented QSSAs in our model of the synthetic predator–prey system by importing the equations into Mathematica and equating the right-hand side of quasi-steady concentrations to zero, and substituting these into the remaining dynamic equations. Consequently, we have achieved only partial automation of this step. We observed that this partial automation circumvented the time-consuming and error-prone nature of conducting algebraic manipulations by hand (§2.5), while maintaining a rigorous derivation of approximate system dynamics. A more complete automation of QSSAs has been implemented in the reb2sac tool, which performs time-scale separation analysis to determine removable states [45]. Incorporating the reb2sac method would enhance our ability to analyse system dynamics and minimize the time it takes to select optimal part configurations for synthetic biological circuits at the cell level.

We used numerical continuation to obtain a deeper understanding of the effects of parameter variation on the non-spatial population dynamics (figure 3 and the electronic supplementary material, figure S1). However, it could also be used to explore the effects of varying important reaction rate parameters on the dynamics of intracellular reactions, or the properties of plausible travelling wave solutions in spatio-temporal simulations for specific parameter values or ranges of parameter values. In particular, identifying the range of possible travelling wave solutions, their wave speeds, wavelengths and stability would help in the design of the spatial domain, helping, for example, to identify a suitable Petri dish size and simulation experiment length in order to obtain the desired spatio-temporal dynamics. Incorporating numerical continuation software such as Auto [46,47] or Wavetrain [48] into our synthetic design framework could therefore be worthwhile.

To develop models at the population level, we used standard formulae for representing resource-dependent birth rates and cell diffusion [30]. Bacterial chemotaxis was omitted for simplicity, although there are also standard formulae for representing this [30]. However, differences between how the loss rate term is typically modelled (linear or density-dependent *per capita* mortality rates, [30]) and other studies highlighting the possible role of induced dormancy [22,23] led us to experiment with different models for the loss rate term. Further clarification of the most appropriate models would be valuable.

Despite this element of uncertainty, modelling *cell* population dynamics for a number of common experimental scenarios can typically be done using standard functions. Theoretical prediction of bacterial spatio-temporal dynamics was recently successfully demonstrated in microfluidic chemostats [35], another potentially useful experimental set-up for automated design. If the behaviour of cell population dynamics can be sufficiently well characterized for a commonly used variety of experimental scenarios, then it is likely that prediction of the spatio-temporal dynamics of such populations could be automated further, allowing automated experimentation and detection of desired population behaviours.

The study of Song *et al.* [21] demonstrated that synthetic predator–prey systems could be used to address questions relating to spatio-temporal dynamics. Our study highlights a natural extension to that work: simulating spatio-temporal dynamics over longer time scales to allow cycles of multiple generations. However, effects resulting from running longer experiments are likely to influence the outcomes of such experiments. One of these is the poorly understood response of cells to density-dependent effects, as discussed earlier. Another is evolution within the cultures leading to the loss of the synthetic functionality. However, if these obstacles can be overcome, then there are a number of further potential uses of synthetic predator–prey systems for research. If such systems could be engineered to reliably generate non-homogeneous spatio-temporal dynamics, then they could be used to directly investigate the influence of various forms of heterogeneity on spatio-temporal dynamics: a topic of widespread scientific interest [29,49]. Moreover, despite existing mathematical theory on the origins of the variations of spatio-temporal dynamics observed in animal populations [24], experimental testing of their predictions has been prohibitively expensive. In such cases, experimental microcosms might be appropriate systems to test theoretical predictions.

One surprising outcome from our study was that the chosen set of parameter values could lead to the generation of dynamical stabilization and spatio-temporal chaos behind the invasion front. Indeed, the phenomenon of an approximately constant-width band in the one-dimensional simulation (figure 5) is so similar to the periodic travelling wave band observed in studies of other oscillatory reaction–diffusion systems under invasion (see [25]) that we hypothesize that it occurs for a related underlying mechanism. Presumably, the small differences remaining from approaching the coexistence steady state behind the invasion front grow in time but with velocities less than that of the invasion front. The width of the visible band is then determined by the unstable mode whose growth rate and velocity determine the shortest distance necessary to achieve a given extent of growth behind the invasion front [25]. Dynamical stabilization in biological systems has only been seen using very abstract models to date [43]. Indeed, studies of complex spatio-temporal dynamics behind invasion fronts in ecological systems have also primarily been restricted to studies of the predictions of theoretical models, without knowledge of whether there exists any ecological system in the real world that truly conforms to the hypothesized mechanisms [24]. Our study indicates that it may be possible to use synthetic ecological systems to generate such dynamics in the laboratory.

Future experiments will be essential not only to test the predictions of our specific case study, but also to demonstrate whether our framework can be used to successfully design real synthetic systems with prescribed population behaviours in practice. Both of these developments would advance synthetic biology as a discipline.

## 4. Methods and mathematical model

### 4.1. Model reduction

Model reduction of the reaction set generated by the GEC software can be automatically condensed into a small set of ODEs, enabling the analysis of the effects of the intracellular synthetic programme on the behaviour of the cell populations. Initially, we derived two single-cell models, one each for the predator and prey cells. We then incorporated these into models for the population behaviours (see the electronic supplementary material, section A.3 and A.4, for detailed analysis of the equations).

Obtaining the same equations as those analysed in Balagaddé *et al.* [8] would justify the use of GEC in designing complex synthetic biological systems. However, our approach identified an error in the original study of Balagaddé *et al.* [8], in which the parameter *K*_2_ is assumed to be IPTG-independent. *K*_2_ is a measure of the sensitivity of the prey cells to the signal (*A*_1_) received from the predators, and incorrectly combines the binding/unbinding characteristics of the signal to the receiver protein LasR and the production/turnover of LasR itself. Since LasR mRNA is transcribed from a genetic construct under the control of P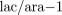 [8], it is necessarily IPTG-dependent, as reflected in our model. This illustrates how using design automation can improve our ability to produce accurate, analysable models from high-level design specifications.

Another difference between our model and that of Balagaddé *et al.* [8] is that we explicitly modelled the lac repressor as a tetramer (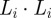), formed as a complex of two lacI dimers *L*_*i*_ that are specifically not bound to IPTG (*I*_*i*_). The complex between a lacI dimer and IPTG is denoted as 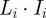. After deriving the quasi-steady states of the system, we found that this scheme for the lac repressor resulted in a different functional form for IPTG dependence than a simple Hill function with exponent 2 (see the electronic supplementary material, equations S7b, S7d, S10d).

Despite the model used in Balagaddé *et al.* [8] not accurately representing the system dependency on IPTG, we chose to perform analysis on their model so that our results are comparable with their study. We keep in mind that, as IPTG levels are increased, there is a progressively smaller effect on *a*_2_ and *K*_2_.

### 4.2. Estimation of maximum carrying capacity, c_*max*_, and the theoretical maximum volume that could be occupied by cells, c_*abs*_

Approximate physical dimensions for *E. coli* are 0.5 µm wide by 2 µm long. Their volume is approximately 0.65 µm^3^. Given these physical dimensions, 1 µm^3^ of volume could theoretically contain about 1.54 cells. This implies that an absolute physical maximum density is 1 540 000 cells per µm^3^ of volume, which is more than an order of magnitude higher than the carrying capacity used by Balagaddé *et al.* [8] of 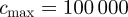 cells *μ*m^−3^. We therefore used 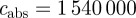 µm^−3^ as the maximum theoretical volume that could be occupied by cells.

The physical maximum of 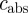 would never be realized in reality; *E. coli* would become resource limited or be influenced by the production of waste before this density is reached and there is also a physical limit to how closely *E. coli* naturally pack (e.g. Volfson *et al.* [35] observed a maximum density of 80% in two-dimensional biofilms). Instead, we explore the effects of increasing 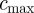 up to 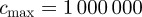 cells, an order of magnitude higher than that used by Balagaddé *et al.* [8] but achieving only up to 65 per cent of the maximum possible packing density.

### 4.3. Parameter values and initial conditions for the population model

We began with the parameter values given in Balagaddé *et al.* [8] to parameterize our various model formulations. The electronic supplementary material, table S1, summarizes the ranges of parameter values that we explored.

To simplify the analysis, we chose to vary parameters that are likely to be most easily modified in the laboratory, to investigate the effects of parameter variation on the model predictions. Balagaddé *et al.* [8] indicated that IPTG can be used to tune the activation of the inserted genetic components; so we chose to vary this parameter. Likewise, total nutrient availability is likely to be straightforward to modify in the laboratory and we explored the effects of varying that parameter though varying 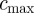. The decay rates of the chemicals 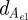 and 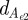 can be affected by the pH of the medium [8], which could also be manipulated in the laboratory. It is plausible that variations in pH may also vary other kinetic factors of *E. coli* population dynamics, though we assumed that this was not the case for our study and varied only 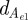 and 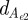. We generally chose parameter value ranges spanning those used by Balagaddé *et al.* [8], within realistic limits.

We assumed that all experiments started with a low initial abundance of predator and prey cells, and that antidote and killer levels corresponded to their equilibrium concentrations. This latter decision was made to regulate the initial dynamics: if no antidote AHL was present initially, then the predator population would take a long time to increase, and, in the absence of killer AHL, the prey population would rapidly increase, depleting resource levels and preventing the predator population from increasing.

### 4.4. Numerical analysis

For some simple formulations of equations (2.1), such as the original formulation of Balagaddé *et al.* [8] with zero dilution, it is possible to derive closed-form expressions for steady states and calculate their linear stability. However, the resulting expressions are typically cumbersome, making them of limited value and, moreover, we cannot obtain closed-form expressions for all formulations considered here. Thus, all of the analyses of equations in this study rely on numerical analysis.

To investigate the predictions of the various model formulations under the various ranges of parameter values, systems of ODEs were solved numerically using the Matlab ode45 routine, which implements an adaptive step-size Runge–Kutta integrator [50]. The integration tolerances were set to provide accurate solutions for concentrations as low as 10^−10^ nM. For each set of parameters, we performed a Fourier analysis to detect population cycles (using fast Fourier transform in Matlab). To remove the initial transient dynamics, we ran the simulation over a period of 2500 hours and then used the last 1024 hours to perform the Fourier analysis. We concentrated on whether our models could predict population cycles over time periods ranging from few days to one or two weeks. A number of our simulations predicted population cycles in which the predator abundances went extremely low, and the period of the oscillations was greater than 1024 hours. We categorized these cases as predator extinction events.

### 4.5. Bifurcation analysis with Auto

Numerical continuation software enables the autonomous monitoring of changes in long-term solutions to systems of ODEs, most commonly equilibrium solutions and periodic orbits, to changes in parameter values. We used numerical continuation, using the software package Auto [46,47], to investigate in detail the bifurcation structure when varying parameter values in each model formulation. This analysis was used to verify that the results obtained were consistent with the period analysis resulting from numerical simulations. We provide more details on the analysis in the electronic supplementary material, section C.
